# Spermatic Cord Sarcoma: A 20-Year Single-Institution Experience

**DOI:** 10.3389/fsurg.2020.566408

**Published:** 2020-11-17

**Authors:** Massimo Iafrate, Giovanni Motterle, Carlotta Zaborra, Niccolò Leone, Tommaso Prayer-Galetti, Filiberto Zattoni, Andrea Guttilla, Rocco Cappellesso, Angelo Paolo Dei Tos, Carlo Riccardo Rossi, Paolo Del Fiore, Marco Rastrelli, Simone Mocellin

**Affiliations:** ^1^Urology Clinic Department of Surgical, Oncological and Gastroenterological Sciences, University of Padua, Padua, Italy; ^2^Clinica Urologica dell'Ospedale di Camposampiero, Camposampiero, Italy; ^3^Surgical Pathology and Cytopathology Unit, Department of Medicine (DIMED), University of Padua, Padua, Italy; ^4^Surgical Oncology Unit, Veneto Institute of Oncology (IOV-IRCCS), Padua, Italy; ^5^Department of Surgery, Oncology and Gastroenterology (DISCOG), University of Padua, Padua, Italy

**Keywords:** soft tissue sarcoma, spermatic cord, spermatic cord sarcoma, paratesticular sarcoma, urologic sarcoma, treatment

## Abstract

**Introduction:** Spermatic cord sarcomas represent a rare genitourinary malignancy with a challenging diagnostic and therapeutic pathway. Different histotypes have been described and prognostic factors remain poorly defined due to the paucity of data presented in literature.

**Methods:** Retrospective chart review of 22 adult patients treated for spermatic cord sarcoma in a single institution in the last 20 years was performed. Clinicopathological characteristics of the tumors were collected with primary and subsequent treatment. Survival analysis was performed in order to identify prognostic factors of disease-specific survival.

**Results:** The median age at diagnosis was 68 years (58–78), the most common histotype was liposarcoma (14/22), and most patients (63.6%) were found to have positive surgical margins after surgery. The 5-year cancer specific survival was 91.3%. Grading (*p* = 0.480), histotype (*p* = 0.327), and type of intervention (*p* = 0.732) were not associated with survival. All patients dead of disease had positive surgical margins (*p* = 0.172).

**Conclusion:** We report a good prognosis at 5 years. Wide radical resection remains the first and probably the most important step; thus, according also to literature, negative surgical margins should be aimed.

## Introduction

Spermatic cord masses represent a challenge in clinical practice, due to their rarity and absence of well-defined, preoperative, diagnostic criteria.

Genitourinary (GU) sarcomas account for <5% of all soft-tissue sarcomas and <2% of malignant urologic tumors (1). Among GU sarcomas, the most commonly reported subtypes are liposarcoma (20–32%), leiomyosarcoma (19–32%), and rhabdomyosarcoma (11–24%). Other rare variants include undifferentiated pleomorphic sarcoma and desmoplastic round cell sarcoma (2). The spermatic cord is the most commonly involved urologic site and is thought to comprise 30–90% of all GU sarcomas. Sarcomas are the most common paratesticular malignant lesion (3) and thus can be grouped, according to location, in the testicular tunica, epididymis, or spermatic cord itself (4).

A bi-modal age presentation for paratesticular sarcomas has been reported for ages 16–20 and >60. Moreover, in the adolescent and young adult population, rhabdomyosarcoma shows the highest incidence, while in the older aged group, liposarcoma, and leiomyosarcoma are the most common subtypes.

The typical presentation is a unilateral inguinal swelling or scrotal mass, which may or may not be painful and is occasionally accompanied by a hydrocele. Occasionally, an initial sign of disease may be acute scrotum due to necrosis or intra-tumoral bleeding (5, 6). Due to non-specific findings, the preoperative distinction between malignant paratesticular tumors and other benign inguinoscrotal conditions such as inguinal hernia, hydrocele, lipoma, hematocele, tuberculosis epididymitis or orchiepididymitis, and malignant lesions of the testis is mandatory even if difficult, in order to avoid potential incomplete resection or contamination of surgical field.

In the presence of a scrotal mass, ultrasonography is the first-line imaging test to characterize the location and, thus, to differentiate intratesticular from paratesticular masses but might fail in distinguishing herniated fat from a lipomatous mass. CT and MRI are useful to evaluate the extent of the disease beyond the inguinal ring and to investigate dimensions, topography, and anatomical relationships of the mass (7).

The final diagnosis is achieved by histologic examination of the mass after surgery or after percutaneous biopsy.

Given the rarity of these tumors, there is little clinical evidence available on which to base multimodal effective treatment strategies.

Radical orchiectomy and wide local resection of surrounding soft tissues has become the accepted standard of management and may be definitive treatment when achieved (8, 9). If the initial surgery is not complete resection, then repeat wide excision is advised.

Several studies have examined the role of adjuvant radiotherapy in STSs of the extremity and have concluded that while it may help in reducing local recurrence, it does not impact overall survival or disease-specific survival (10). The role of chemotherapy in adult GU sarcomas remains controversial. Meta-analysis of studies regarding doxorubicin-based adjuvant chemotherapy has shown similar results with respect to decreasing local and even distant recurrence; however, its effect on overall survival, while beneficial, was not statistically significant (11). Given the dearth of studies and lack of strong evidence, these patients undergo diverse treatment strategies that likely reflect the preference or experience of the treating physician and/or institution.

We already presented as a case series our initial experience with spermatic cord sarcoma (12). In this study, we present an update of our previously published case series with an increased 20-year experience and we identify risk factors for disease-related mortality.

## Materials and Methods

Retrospective analysis of clinico-pathological characteristics and surgical outcomes for 22 adult male patients (>18 years of age at the time of diagnosis) treated for primary or recurrent spermatic cord sarcoma from 1996 to 2018 in Padua was performed. All cases were reviewed by a pathologist, and the diagnoses were confirmed and updated to the latest edition of the World Health Organization classification of soft tissue and bone tumors.

Data abstracted included age, side of the tumor, date of surgery, histopathologic subtype, other tumor characteristics (grade and stage), site and date of recurrence, additional treatment, and follow-up time. Grading was evaluated according to the Federation Francaise des Centers de Lutte Contre Cancer (FNCLCC) system and staging was assigned according to the American Joint Committee on Cancer (AJCC) TNM staging system. Resections were classified according to the pathological specimen as R0 or R1 depending on the presence of tumor within 1 mm from the inked margin.

The study endpoint was cancer-specific survival, and causes of death unrelated to sarcoma were censored.

Distributions were summarized using frequencies, medians, and interquartile range. Cancer-specific survival rates were estimated using the Kaplan–Meier method and with log-rank test. Given the low number of events, no multivariate analysis was performed. Any *p* < 0.05 was considered statistically significant. All analyses were performed using R Statistical Software (Foundation for Statistical Computing, Vienna, Austria).

Ethics approval was not required because, for the study, we used existing data collections and records that contain only unidentifiable human data.

Furthermore, for all retrospective studies involving the only use of anonymized clinical data, the Italian legislation does not provide for the approval of an ethics committee.

## Results

### Patient Characteristics

A cohort of 22 patients with complete follow-up data was analyzed and summarized in Table 1. The median age at diagnosis was 68 years (range, 58–78); the greatest proportion of patients (72.7%) was treated with radical orchifuniculectomy, while the remaining underwent marginal resection of the mass, which, in one case, was an incidental finding during hernioplasty.

**Table 1:** Clinicopathological characteristics of the cohort.

Age at diagnosis, median (IQR)	68 (58–78)
Side, *n* (%)	
- Left	14 (63.6)
- Right	8 (36.3)
Surgery, *n* (%)	
- Orchifuniculectomy	16 (72.7)
- Marginal resection	6 (27.2)
Histotype, *n* (%)	
- Well-differentiated liposarcoma	10 (45.4)
- Dedifferentiated liposarcoma	5 (22.7)
- Leiomyosarcoma	3 (13.6)
- Other	4 (18.1)
Tumor grade, *n* (%)	
−1	9 (40.9)
−2	3 (13.6)
−3	9 (40.9)
Missing	1 (4.5)
Margins, *n* (%)	
- R0	7 (31.8)
- R1	14 (63.6)
Missing	1 (4.5)
Staging, *n* (%)	
- Ia	6 (27.2)
- Ib	4 (18.1)
- IIa	2 (9.0)
- IIb	2 (9.0)
- III	6 (27.2)
- IV	1 (4.5)
Missing	1 (4.5)
Additional treatments, *n* (%)	
- Radiation therapy	2 (9.1)
- Chemotherapy	3 (14.3)
- Second surgical resection	4 (18.2)
- None	13 (59.1)
Follow-up (months), median (IQR)	66.6 (18.4–109)

Three patients had adjuvant chemotherapy, one patient had adjuvant radiation therapy, and four patients had second surgery. Five patients were treated at our institution after a recurrence of the primary disease; thus, we were unable to retrieve complete staging information for one of these patients.

The most common histotype was liposarcoma; in particular, 10 patients had well-differentiated liposarcoma and 5 patients had dedifferentiated liposarcoma. Leiomyosarcoma was found in three cases and four patients had other histotypes, namely, mixofibrosarcoma, epitheliod sarcoma, rhabdomyosarcoma, and one undifferentiated pleomorphic sarcoma. Most patients (63.6%) were found to have positive surgical margins after surgery and 9 (40.9%) presented with G3 disease.

Seven patients overall received perioperative therapy. Three patients performed preop/therapy with neoadjuvant intent: one patient (myxofibrosarcoma G2 stage IIb) performed radiotherapy, and two patients (Epithelioid sarcoma G3 stage III, liposarcoma G3 stage Ib) received chemotherapy. Four patients received postop/therapy with adjuvant intent: two did CT (Liposarcoma G3 stage III, Liposarcoma G3 stage IV) and two did RT (Liposarcoma G1 stage IB, Liposarcoma G1 stage IIB).

In the context of multidisciplinary treatment, radiotherapy has the role of providing local control of the disease. Preop/radiotherapy is indicated in patients in whom conservative surgery cannot be performed at the time of diagnosis due to the size of the disease, the site of onset, or its close proximity to important structures such as bones, vessels, or nerves. Postop/radiotherapy is indicated in the most aggressive tumor forms in order to reduce the risk of a local recurrence and when we have R1–R2 margins. In the case of localized disease, however, it can be used in the pre-operative phase to reduce the size of the primary tumor or in the post-operative phase in the presence of very aggressive forms, to reduce the risk of local recurrence and/or spread of the disease at a distance (Table 2).

**Table 2:** Clinicopathological characteristics of the cohort.

**Intent**	**Type of treatment**	**Histotype**	**Stage**	**Grading**	**R**
Adjuvant	CT	Liposarcoma	III	G3	R1
Adjuvant	CT	Liposarcoma	IV	G3	R1
Neoadjuvant	CT	Epithelioid sarcoma	III	G3	NA
Neoadjuvant	CT	Liposarcoma	IB	G3	NA
Adjuvant	RT	Liposarcoma	IB	G1	R1
Neoadjuvant	RT	Mixofibrosarcoma	IIB	G2	NA
Adjuvant	RT	Liposarcoma	IIB	G1	R1

Five patients overall had recurrence; four patients revealed distant metastases (two had systemic CT treatment and two had none; one patient had a local skin recurrence that was surgically removed).

According to the American Joint Committee on Cancer (AJCC) TNM staging system, one patient with undifferentiated pleomorphic sarcoma presented with metastatic disease (Stage IV) and died of disease after 3 months from the diagnosis.

### Cancer-Specific Survival

**Figure 1:**
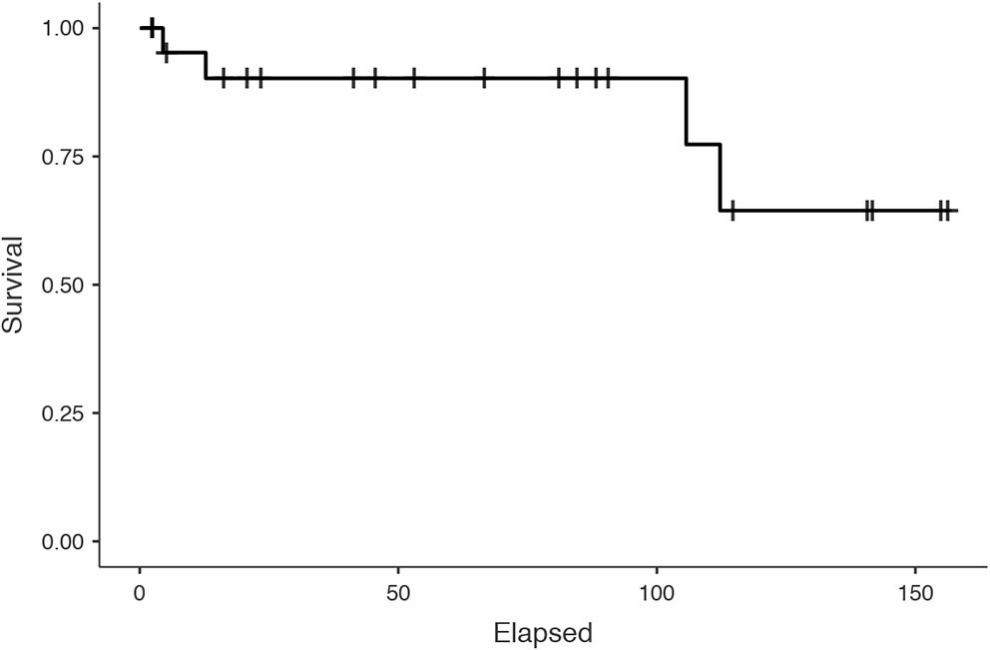
Cancer-specific survival of the whole cohort of patients.

The median follow-up of the whole cohort was 66.6 months (18.4–109). Four patients were dead of disease at the end of follow-up. The 5-year CSS was 91.3% (Figure 1).

**Figure 2:**
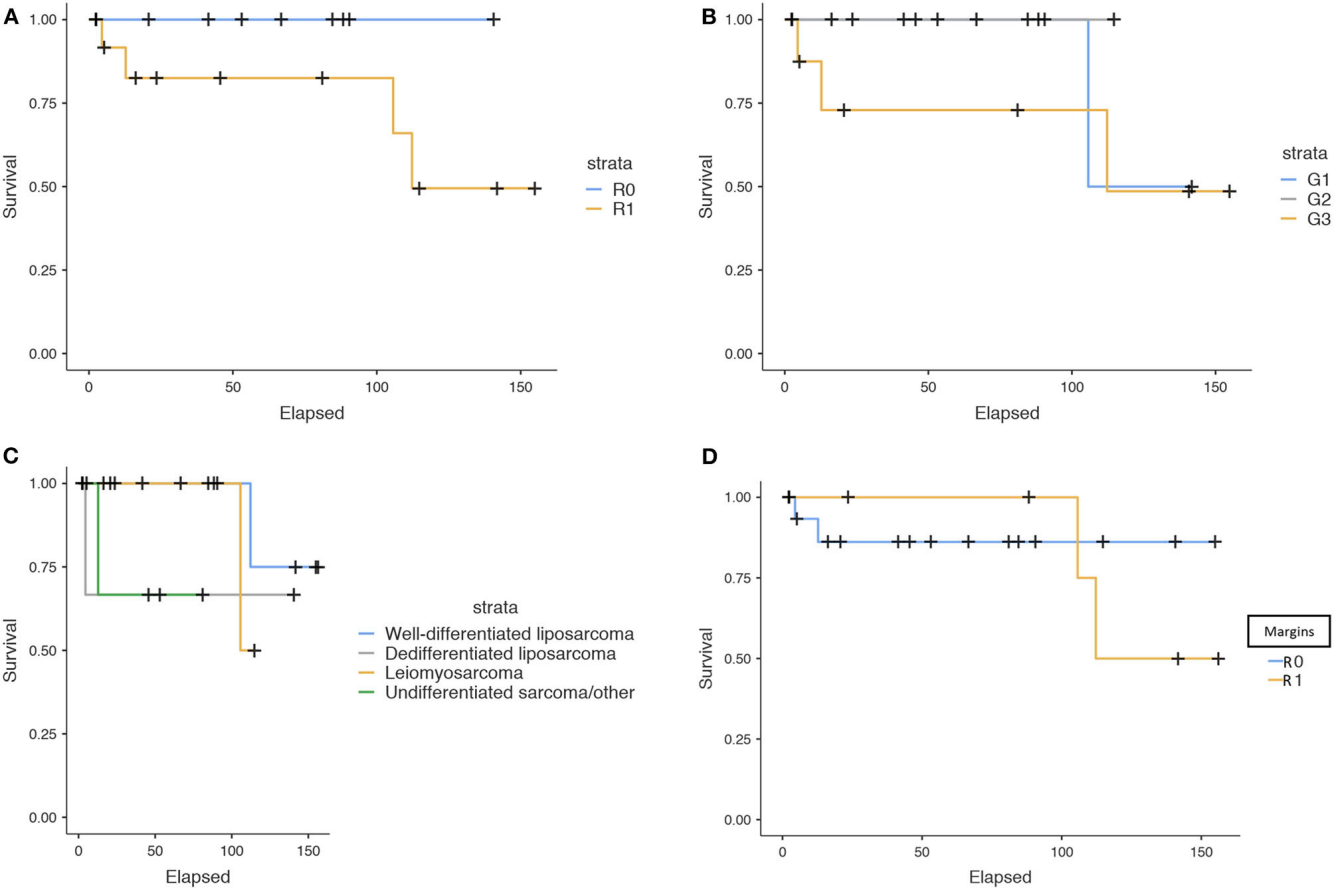
**(A–D)** Cancer-specific survival according to subgroups.

**Figure 3:**
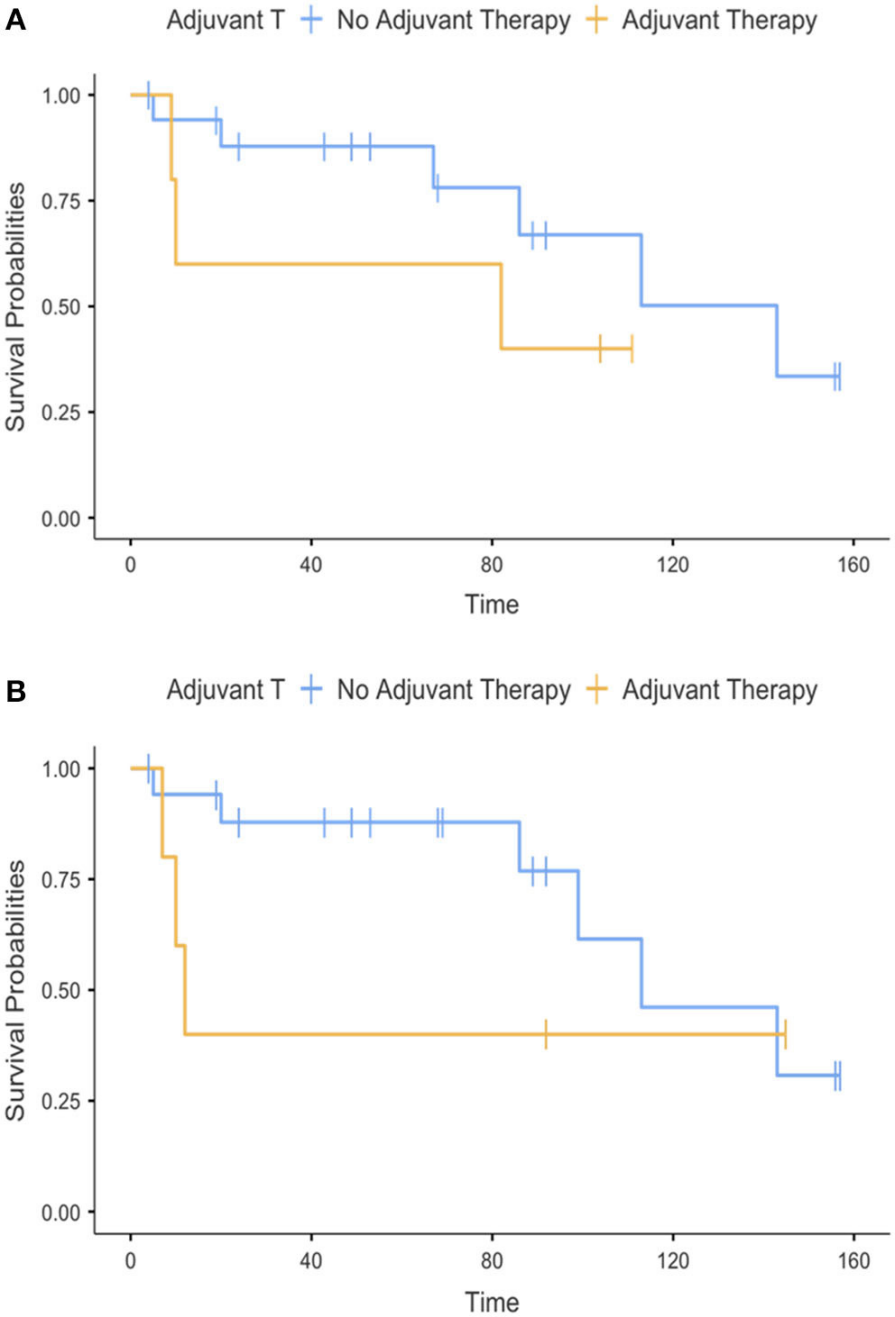
**(A,B)** Cancer-specific survival according to treatments.

CSS survival was analyzed in relationship with the type of intervention, margin status, grading, histotype, and adjuvant therapy and illustrated in Figures 2A–D, 3A,B.

Grading (*p* = 0.480), histotype (*p* = 0.327), type of intervention (*p* = 0.732), and adjuvant therapy (*p* = 0.2075) were not associated with CSS. However, it is noteworthy that all patients dead of disease had positive surgical margins (*p* = 0.172), while none of those with negative margins died of disease.

## Discussion

Spermatic cord sarcomas, although rare, are highly malignant tumors that are best managed with radical surgery and may require additional treatment according to their staging (13). It must be taken into consideration that many cases could be initially misdiagnosed and radical orchiectomy might not have been discussed preoperatively with the patient as an option, thus increasing the risk of an incomplete resection of the tumor. Management of spermatic cord sarcomas is challenging because of the lack of high-quality data in the literature and specific guidelines. Only three patients with positive surgical margins had no imaging performed pre-operatively, two patients had CT-only staging and seven patients had a combination of US, CT, and MRI. These results could be an indicator of inaccuracy of the imaging technique or inadequate surgical planning. Five of our patients were treated in other institutions with a non-radical intervention, and four of them underwent a second resection for disease recurrence or persistence, possibly reflecting this common scenario. It is likely that variability in the management of the primary tumor further extends the complexity of adjuvant and salvage treatments, thus increasing the complexity of these clinical cases.

Many studies have investigated GU sarcomas and tried to investigate predictors of recurrence and survival. Within 1,583 sarcomas studied by Russo et al. (14) tumor size (<5 cm), low histologic grade, and complete surgical resection were favorable prognostic indicators of survival. Notably, only 14 paratesticular sarcomas were included in this cohort. Dotan et al. (9) presented an extended cohort with longer follow-up, eventually identifying 57 sarcomas of paratesticular origin. After multivariate analysis, they reported that tumor size and absence of metastasis at diagnosis were the only significant predictors of disease-specific survival.

Additionally, Stojadinovic et al. (15) reported that positive surgical margins significantly increased the risk of local recurrence (28 vs. 15%, *p* < 0.001) in addition to increasing the risk of distant metastases and disease-related death.

Wang et al. (16) performed an analysis of predictors of survival in a cohort of 188 adult patients with GU sarcomas. In a multivariate analysis, they reported that patient age <50 years and incomplete surgical resection were both predictors of recurrence-free and overall survival.

Our study represents one of the largest and most homogeneous cohort studies of patients with spermatic cord sarcomas, since it is the experience of a single center dedicated multidisciplinary team. Our findings, although not significant, are interesting, and somewhat consistent with previous studies.

First of all, we present a cohort with one of the longest median follow-up time reported in literature, exceeding 5 years. Within this follow-up time, we found a 5-year CSS 91.3%, providing evidence of good prognosis for this disease, when adequately managed, even in case of a potential incomplete first resection.

We found a non-significant trend of relationship between positive surgical margins and disease-specific mortality (*p* = 0.172). Every patient who died for disease had positive surgical margins, while none of those with negative margins died of disease. Unfortunately, given the low number of events, the test had not enough statistical power to provide a significant *p*-value. We were unable to demonstrate a relationship between the type of surgical intervention performed and survival; this corroborated the idea that surgical resection should aim for excision of the tumor and surrounding tissues until negative margins are obtained.

The need for a wide resection with negative surgical margins is consistent with findings of Goldberg et al. (17) where hemiscrotectomy, both primary and completion, was associated with lower local recurrence rates (HR 0.21, *p* = 0.02) and overall survival (*p* = 0.081).

Similarly, median survival for the patient with metastasis at diagnosis was very poor, emphasizing the need for prompt and accurate diagnosis and treatment. Our experience is consistent with the reported epidemiology, where liposarcoma was the most common histotype (15/23), while rhabdomyosarcoma was present only in one 18-year-old patient.

Our study included adjuvant therapies; however, the diversity of radiation and chemotherapy regimens along with the small sample size prohibited any analysis regarding these modalities.

In the multicenter study by Radaelli et al. (18) the quality of surgical margins was associated with local recurrence (*p* = 0.025) and disease-specific survival, especially in the liposarcoma subgroup (*p* = 0.043).

There are several limitations in our study; most of them are explained by its retrospective design and by the small data set that did not allow for multivariate analysis. Moreover, adjuvant treatments were individualized, and their potential therapeutic effect could not be discriminated from a selection bias related to those with unfavorable pathologic features. Unfortunately, these are limitations shared with the other mentioned studies, and the rarity of these tumors still makes them the only evidence available. Likely, only a collaborative international effort to gather together the clinico-pathological data on these rare tumors might at least in part overcome these limitations.

Despite its limitations, this remains one of the largest single-center studies to date and with a considerably long follow-up, related specifically to spermatic cord sarcomas. This specific disease, given its location and atypical presentation, should be distinguished from other GU sarcomas.

## Conclusions

In conclusion, spermatic cord sarcomas are uncommon tumors with a challenging diagnostic and therapeutic pathway. Our experience showed a good prognosis at 5 years, with most of the localized tumors that can be cured by surgical resection. According to our experience and literature, margin status remains of outmost importance in determining the prognosis; thus, effort should be put in obtaining complete excision of the tumor. We must underline that, most of the time, the first physician who encounters these patients is the urologist, who is usually not familiar with sarcomas. In this setting, with the conclusions of this manuscript, we hope to raise the suspicion of this disease in case of spermatic cord masses, to emphasize the importance of radical intervention, and to provide an overview of the prognosis. It is undoubtable that treatments and prognosis at the individual level should be based on a multidisciplinary discussion. Further studies are required to elucidate optimal adjuvant therapy and appropriate patient selection for it.

## Data Availability Statement

The raw data supporting the conclusions of this article will be made available by the authors, without undue reservation.

## Ethics Statement

The research was conducted ethically in accordance with the World Medical Association Declaration of Helsinki. The ethical review of human research was not required because, for the study, we used existing data collections and records that contain only unidentifiable human data.

## Author Contributions

MI and SM designed clinical studies. CZ, NL, AG, RC, and PD analyzed the data. MI, SM, and MR wrote the manuscript. TP-G, FZ, and CR performed surgery. MI, AD, and SM reviewed, revised, and approved the final paper. All authors contributed to the article and approved the submitted version.

## Conflict of Interest

The authors declare that the research was conducted in the absence of any commercial or financial relationships that could be construed as a potential conflict of interest.
